# The Immunology of Syncytialized Trophoblast

**DOI:** 10.3390/ijms22041767

**Published:** 2021-02-10

**Authors:** Danny J. Schust, Elizabeth A. Bonney, Jun Sugimoto, Toshi Ezashi, R. Michael Roberts, Sehee Choi, Jie Zhou

**Affiliations:** 1 Department of Obstetrics, Gynecology, University of Missouri School of Medicine, Columbia, MO 65202, USA; toshiko.ezashi@missouri.edu (T.E.); robertsrm@missouri.edu (R.M.R.); seheechoi@missouri.edu (S.C.); zhojie@health.missouri.edu (J.Z.); 2 Department of Obstetrics, Gynecology and Reproductive Sciences, University of Vermont College of Medicine, Burlington, VT 05405, USA; Elizabeth.bonney@med.uvm.edu; 3 Department of Obstetrics and Gynecology, Hiroshima University, Hiroshima 734-8551, Japan; juns@hiroshima-u.ac.jp; 4 Division of Animal Sciences, University of Missouri, Columbia, MO 65211, USA; 5 Christopher S. Bond Life Sciences Center, University of Missouri, Columbia, MO 65211, USA

**Keywords:** syncytiotrophoblast (STB), toll-like receptors (TLRs), immune checkpoint molecules, extracellular vesicles (EV), human endogenous retroviruses (HERVs)

## Abstract

Multinucleate syncytialized trophoblast is found in three forms in the human placenta. In the earliest stages of pregnancy, it is seen at the invasive leading edge of the implanting embryo and has been called primitive trophoblast. In later pregnancy, it is represented by the immense, multinucleated layer covering the surface of placental villi and by the trophoblast giant cells found deep within the uterine decidua and myometrium. These syncytia interact with local and/or systemic maternal immune effector cells in a fine balance that allows for invasion and persistence of allogeneic cells in a mother who must retain immunocompetence for 40 weeks of pregnancy. Maternal immune interactions with syncytialized trophoblast require tightly regulated mechanisms that may differ depending on the location of fetal cells and their invasiveness, the nature of the surrounding immune effector cells and the gestational age of the pregnancy. Some specifically reflect the unique mechanisms involved in trophoblast cell–cell fusion (aka syncytialization). Here we will review and summarize several of the mechanisms that support healthy maternal–fetal immune interactions specifically at syncytiotrophoblast interfaces.

## 1. The Hemochorial Placenta Poses Unique Immune Challenges

Humans are grouped with most, but not all non-human primate species and rodents, including rats, mice, and guinea pigs, by their shared use of a hemochorial form of placentation. As the name would suggest, maternal blood comes into direct contact with fetally-derived placental cells (trophoblast) in the hemochorial placenta. This form of placentation is also characterized by fairly deep invasion of trophoblast cells into maternal uterine tissues, although the human stands out in this regard, with trophoblast cells found throughout the endometrial decidua and even in the myometrium [1]. These uterine decidua and myometrium are populated by maternal immune cells and close contact, here and elsewhere (see below), between fetally-derived cells and maternal immune cells poses a potential allogeneic threat in a mother who must still combat invading pathogens. This conundrum has been extensively studied since first proposed [2]. To summarize these many years of investigation is beyond the purview of this review. Suffice it to say that the immunologic interactions that allow stringent maintenance of maternal pathogen protection in order to avert infections, which could either interrupt pregnancy or be transmitted vertically to the fetus, yet support the mandate to tolerate semi-allogenic fetal tissues through 9 months of pregnancy are complex, nuanced and tightly regulated [3]. They are most certainly not characterized by simple protolerogenic responses.

**Figure 1:**
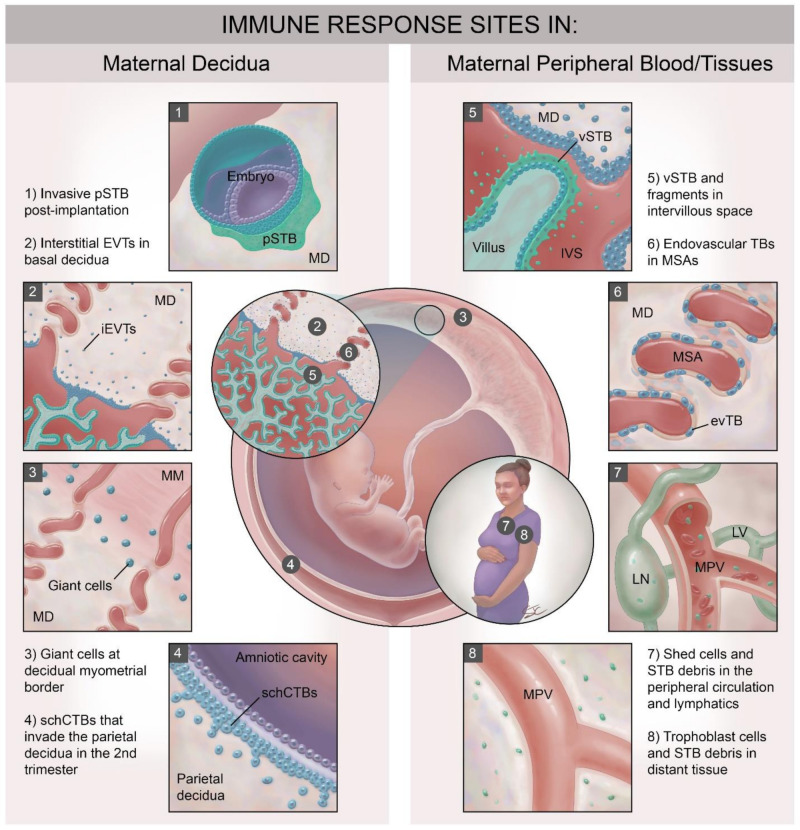
Sites of interaction between fetal-derived trophoblast and maternal immune cells in the pregnant woman. Trophoblast-derived material, entire cells and the multinucleated syncytiotrophoblast layer encounter maternal immune cells at no less than eight main sites. These can be divided into those that interact with systemic maternal immune cells in peripheral blood and peripheral tissues and those that encounter the specialized immune cell populations in the maternal decidua. EVT: extravillous trophoblast; evTB: endovascular trophoblast cells; iEVT: interstitial EVT; IVS: intervillous space; LN: lymph node; LV: lymphatic vessel; MD: maternal decidua; MM: maternal myometrium; MPV: maternal peripheral vessel; MSA: maternal spiral artery: pSTB: primitive syncytiotrophoblast, schCTBs: smooth chorionic cytotrophoblast cells; vSTB: villous syncytiotrophoblast.

In the first few days after implantation, the human placenta develops from the trophectoderm layer of the blastocyst as a primitive syncytium that invades the maternal decidua. This primitive syncytium is unique in that it both (1) secretes the large amount of human chorionic gonadotropin (hCG) necessary to support the corpus luteum and (2) acts as the leading edge of the invasive placenta. By several weeks of pregnancy, the placenta has changed via fusion of intercellular fluid filled spaces or lacunae so that tree-like villous structures containing fetal vessels and stroma are floating in mainly maternal glandular secretions that fill the intervillous space between the maternal decidua and the placental villi (Figure 1). Some villi extend across the intervillous space and remain attached to the maternal decidua as anchoring villi. Those villi that completely “float” in the intervillous space are covered by two layers of trophoblast cells—an inner layer of proliferative cytotrophoblast cells (CTB) and an outer single layer of fused cytotrophoblast cells called the syncytiotrophoblast (STB) across which maternal nutrients and fetal wastes pass. As pregnancy progresses, the STB layer is under constant turnover, incorporating new CTB and shedding debris—extracellular vesicles and even large syncytial knots—into the intervillous space, where they can ultimately enter that maternal circulation. While the lateral edges of the anchoring villi are similarly covered by outer STB and inner CTB layers, the tips of the anchoring villi are populated by villous cytotrophoblast progenitors that transform into invasive extravillous trophoblast cells (EVT). EVT move through and along the maternal decidua to become: (1) endovascular trophoblast cells that transform their invasive capacity and adopt a vascular phenotype as they remodel maternal spiral arteries [4,5], (2) interstitial EVT that populate the maternal decidua and invade into the maternal myometrium without significant fusion and (3) fusion-mediated multinucleated trophoblast giant cells (TGC) that can be found mostly near the maternal decidual/myometrial border. While most believe end differentiated interstitial EVT fusion to be the origin of TGC, other cellular origins for this giant cell type have been posited.

Strategies to balance the immunologic imperatives of pathogen protection with allograft acceptance at the human maternal–fetal interface likely differ depending on the specific site of maternal immune mediator/trophoblast subtype interaction and upon gestational age. For instance, while interstitial invading EVT cells encounter the unique local immune cells populating the maternal decidua, STB contacts mainly endometrial glandular secretions and peripheral blood plasma exudates early in pregnancy [4]. Later in pregnancy, with full remodeling of the maternal spiral arteries and dissolution of the spiral artery plugs (completed around twelve weeks gestation) [4], endovascular trophoblast and the STB encounter the totality of cellular and soluble immune components of maternal peripheral blood.

Over the time course of human pregnancy, placental trophoblast cells and maternal immune mediators interact at a minimum of eight major sites (Figure 1), (also reviewed elsewhere in this issue [5]). Four of these sites involve interactions with specialized [6,7] immune cells populating the maternal decidua, many of which are already present in the post-ovulatory decidua just prior to blastocyst attachment (Figure 1, panels 1–4). Trophoblast subtypes that interact with decidual immune cells include: (1) the post-implantation invasive primitive syncytium of the implanting conceptus (pSTB, Figure 1, panel 1); (2) the interstitial EVT populating the basal decidua (Figure 1, panel 2); (3) the trophoblast giant cells at the decidual–myometrial border (Figure 1, panel 3) and (4) the smooth chorionic cytotrophoblasts (schCTBs) that invade into the parietal decidua (Figure 1, panel 4) [8]. These trophoblast subtypes share expression of a subset of somewhat unusual major histocompatibility complex (MHC) class I molecules [9,10,11] that allow communication with the specialized and unique decidual immune cells. Interestingly, even within the decidua, maternal immune cell populations differ in quantity and phenotype between the basal and parietal sites [12]. Four maternal immune cell/trophoblast interfaces in humans involve interactions between fetally-derived trophoblast cells, trophoblast debris and trophoblast-derived microvesicles and the cellular and soluble immune mediators present in the maternal blood (Figure 1, panels 5–8). These sites include: (1) at the apical surface of the intact villous syncytiotrophoblast (vSTB; Figure 1, panel 5), (2) within the remodeled maternal spiral arteries (MSA), where endovascular trophoblast cells line the lumen (Figure 1, panel 6), and (3) in the intervillous space and in the maternal peripheral circulation and lymphatic system where shed trophoblast debris and cells circulate (Figure 1, panels 5 and 7). Finally, STB debris and even entire fetal trophoblast cells can be detected in distant maternal tissues during pregnancy and even many years after delivery (Figure 1, panel 8) [13]. The latter phenomenon is known as fetal microchimerism [14], Presumably these shed trophoblast-derived cells and other material will interact with local tissue resident immune cells. Fetal microchimerism has been associated with several autoimmune disorders [15], with wound healing [16], and with transgenerational health [17], but will not be reviewed further here. In this review, we will concentrate on immune interactions with syncytialized trophoblast and focus largely on the interactions between: (1) maternal peripheral immune cells (peripheral blood mononuclear cells or PBMCs,) and vSTB (Figure 1, panels 6 and 7) and (2) between decidual immune cells and primitive STB (Figure 1, panel 1) and trophoblast giant cells (TGCs Figure 1, panel 3).

## 2. Immune Interactions at STB Interfaces

It remains unclear whether the unique immune characteristics of STB at any of its sites are inherent to the differentiation of CTB prior to syncytialization, the fusion process itself, the mechanisms involved in maintaining a functioning vSTB or whether they occur by other mechanisms. Here we will describe several of these immune processes and hypothesize on their origins and mechanisms.

### 2.1. A Possible Role for Glycosylation

The multinucleated, single layer of vSTB is one of a very limited number of human cellular entities that essentially lack MHC class I and MHC class II antigen presenting molecules [3,18,19]. An absence of the latter is not particularly surprising as MHC class II expression is typically restricted to antigen presenting cells. The mechanism by which non-immune cells [18,19], including trophoblast cells [20], limit such expression has been described. MHC class I molecules, however, are typically expressed on the surfaces of all somatic cells to mediate immune cell recognition of intracellular pathogens, neoplastic transformation, and dysregulated cellular homeostasis. The mechanisms mediating this unusual lack of MHC expression on trophoblasts remain unclear [21,22]. While the complete absence of MHC class I molecules on the surface of the vSTB and villous CTB layer of the human placenta likely aids in escape from alloimmune recognition; such lack of expression of crucial informational macromolecules may make such trophoblast susceptible to lysis by certain types of peripheral natural killer (pNK) cells in the maternal circulation [23], depending on pNK cell phenotype and function.

Mechanisms for protection from susceptibility to lysis are not fully elucidated, but characteristic surface glycosylation patterns on STB and villous CTB (vCTB) may be involved. The hypothesis here is that the glycans act as a shield from NK cell-mediated cytotoxicity, and, although less studied, other adverse interactions with immune cells. Although the exact NK cell surface receptors for glycan-mediated suppression have yet to be defined, one or more of the sialic acid-binding immunoglobulin-type lectins known as Siglecs, including Siglec 9, are enticing candidates [24].

vCTB and vSTB express both high mannose and complex-type glycans [25]. Most of the complex type glycans are core fucosylated biantennary types, many of which express a bisecting N-acetylglucosamine (GlcNAc) [26]. These N-glycans have been associated with the suppression of pNK cell cytotoxicity in vitro [27,28]. Substantial expression of biantennary bisecting type N-glycans on STB may help to explain the notable and somewhat unexpected resistance of STB to pNK cell-mediated cytotoxicity in vitro. Since both vCTB and vSTB express similar glycan expression patterns, it can be hypothesized that these glycan decorations are “carried over” during syncytialization at the villous surface but are not strictly involved in the syncytialization process itself, nor in its maintenance.

Alterations in trophoblast glycosylation and glycan processing have been linked to several pregnancy disorders, including miscarriage, preeclampsia and intrauterine growth retardation, and such alterations have been demonstrated to have immune underpinnings [29,30]. For example, villous trophoblast proteoglycans in pregnancies complicated by preeclampsia lacked decoration by acetylated sialic acid side chains when compared to placentas from uncomplicated pregnancies [31].

Little is known about the glycosylation status of human trophoblast giant cells or primitive STB, but since these STB populations interface mainly with decidual NK (dNK) cells that differ from pNK cells in phenotype and killing function [32], resistance to lysis may be more importantly a function of the effector cell rather than the target. Alternatively, one or more of the glycoprotein hormones secreted by the trophoblast giant cells (e.g., human chorionic gonadotropin (hCG); human placental lactogen (hPL)) [33] and primitive STB (e.g., hCG) may alter local immune responses via glycan–ligand interactions. Consistent with this concept, it was recently shown that hCG isolated from the urine of pregnant women is decorated with bisected N-glycans, the majority of which are biantennary [34]. Human CG at levels well below those seen in the maternal circulation during early pregnancy have been shown to effectively inhibit the cytotoxic activities of peripheral NK cells against NK-sensitive K562 erythroleukemia cells in vitro [35].

### 2.2. Toll-Like Receptors (TLRs) and STB Immune Responses

For blood-borne pathogens, the vSTB layer is the initial barrier to transplacental transmission and innate immunity, the first defense mechanism. Trophoblast can recognize and respond to microorganisms and their products through the surface expression of toll-like receptors (TLRs) [36,37,38], although expression again varies by cell type and gestational age. TLRs are innate immune sensors for danger signals from infections and damaged tissue and signaling through TLRs typically stimulates inflammation. Ten TLRs (TLR1 to TLR10) and one pseudogene (TLR11) have been identified in humans. TLRs recognize specific pathogen-associated molecular patterns (PAMPs) expressed on invading pathogens and damage-associated molecular patterns (DAMPs) released by cells undergoing damage or apoptosis [39]. TLRs’ recognition and binding to these ligands induces TLR signaling that regulates the expression of a variety of genes encoding cytokines, chemokines, MHC products and co-stimulatory molecules involved in the innate immune response.

Specific TLR expression patterns in the placenta appear to vary by gestational age. In the first trimester, TLR1–8 and TLR10 are expressed in human villous placental trophoblast cells, with expression essentially limited to the villous CTB [40,41]. EVTs and CTB cells highly express TLR-2 and TLR4 in the first trimester of pregnancy, but vSTB lacks such TLR expression [42]. While this lack of TLRs may allow vSTB to avoid overly robust responses to low level pathogen exposures, the expression of TLRs in the underlying vCTB, which, unlike later in pregnancy, remains largely continuous in the first trimester of pregnancy, would allow for robust defense should more serious pathogen invasion or danger signals arise. Further, first trimester vSTB may be less likely to encounter blood-borne maternal pathogens prior to the erosion of decidual spiral artery plug and full exposure to maternal blood. In the second trimester all TLRs, i.e., 1–10, are expressed in trophoblast cells, although not all have well-delineated cell subtype expressions patterns reported [40,41]. At this time in pregnancy, TLR-2 and TLR-4 are localized throughout the placenta in CTB, the vSTB and EVT [43,44,45,46], findings consistent with gestational age dependent responses of trophoblast to pathogens or damage, as exemplified by gestational age specificity to infection as described below.

Normal term placental tissue has been shown to express TLRs 1–10 [40], but TLR2 and TLR5 transcripts appear to increase preferentially in association with labor [47]. Cultured CTB and STB cells isolated from term placenta express TLRs 2- 6 and TLR9 [48,49]. Although some or most these findings could result from the artificial conditions under which the models are propagated; if true, they support the concept that trophoblast cells, including vSTB from the second trimester and third trimester, are able to recognize and respond to microorganisms present at the maternal–fetal interface and initiate immune responses. Whether these responses can control transmission of infection to the fetus without initiating preterm labor likely depends on the identity of the pathogen and the inoculum of microbe or degree of tissue damage. Highlighting their likely importance in health and disease, placentally-expressed TLRs, including those expressed specifically on syncytialized trophoblast [50] have recently been linked to the pathophysiology of preeclampsia [50,51] and early spontaneous pregnancy loss [52].

### 2.3. STB and Immune Checkpoint Molecules

Cancer cells and trophoblast share many common features, including an ability evade immune destruction. One means whereby cancer cells protect themselves is to utilize a strategy in which a cancer cell either directly or indirectly, e.g., through the intervention of dendritic cells, interacts with so-called immune checkpoint proteins on the surfaces of immune cells. The net result of this interaction is to compromise the effector functions of the immune cells by causing atypical signaling through the T cell receptor (TCR), upregulation of immuno-suppressive signaling pathways and, in some cases, T cell exhaustion [53]. Interactions between the “foreign” STB and T cells may offer similar opportunities for immune regulation on the basis of “checkpoints” as occurs in cancer.

Since STB do not express MHC molecules, first exposure to any antigens expressed by normal or distressed/infected STB in the context of MHC would more likely have to happen indirectly, through processing and presentation of “foreign” antigen by dendritic cells in the maternal circulation or the decidua. This alone decreases the efficiency of adaptive responses against such STB antigens. Furthermore, interaction between STB and antigen presenting cells may result in (down)-regulated antigen presenting cell function and, accordingly, altered activation and development of T cell responses upon subsequent interaction between “regulated” antigen presenting cells and T cells. Moreover, a triad of interactions, e.g., naïve T cell, antigen presenting cell and STB may result in altered T cell activation or development due to expression of immune “checkpoint” molecules on STB.

**Figure 2:**
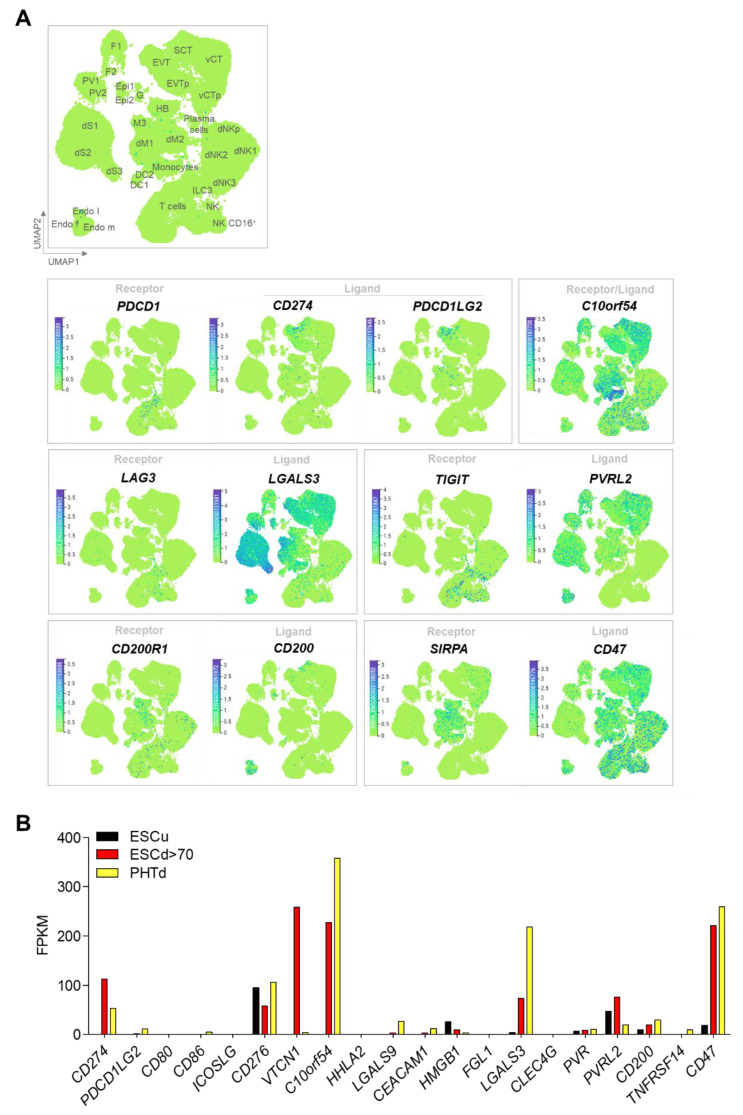
Expression of inhibitory immune checkpoints. (**A**) Uniform manifold Approximation and Projection (UMAP) visualization of the log-transformed, normalized expression of immune checkpoint receptors and ligands in placental and decidual cell clusters of the first trimester. Data are available online at http://data.teichlab.org (maternal–fetal interface) (accessed on 11 November 2018) as provided by Vento-Tormo et al. [58]. DC, dendritic cells; dM, decidual macrophages; dS, decidual stromal cells; Endo, endothelial cells; Epi, epithelial glandular cells; F, fibroblasts; HB, Hofbauer cells; PV, perivascular cells; SCT, syncytiotrophoblast; VCT, villous cytotrophoblast; EVT, extravillous trophoblast; f, fetal; ILC, innate lymphocyte cells; l, lymphatic; m, maternal; p, proliferative; M3, maternal macrophages; G, granulocytes. (**B**) Comparative expression of immune checkpoint inhibitors ligands in undifferentiated H1 ESC (ESCu, black), STB generated from defined DME/F12/KOSR medium that contained BMP4, A83-01, and PD173074 (BAP) treatment-differentiated H1 ESC (ESCd > 70, >70 um size fraction from BAP-differentiated H1 ESC, red), and STB generated from term placenta (PTHd, yellow). Transcript levels are shown in FPKM (Fragments Per Kilobase of transcript per Million mapped reads). RNAseq data are from Yabe et al. [59].

One of the best characterized immune checkpoints, and one of two unequivocally shown to operate in cancer cells, involves the interaction between programmed cell death-1 (PDCD1, often known as PD1) on the T cell and its ligand (CD274, often known as PD-L1). In first trimester and term human placenta, CD274 is preferentially expressed in vSTB compared to vCTB, suggesting that vSTB can inhibit activation of maternal peripheral T cells [54]. To that point, soluble CD274 is higher in the sera of pregnant women compared to non-pregnant controls and sera-induced activation of mixed lymphocyte cultures can be blocked by a CD274-blocking antibody [55].Others have demonstrated by western immunoblotting that placenta CD274 expression increases dramatically with the onset of the second trimester, when full flow through the maternal spiral arteries has been fully established [56]. vSTB in early human placenta also exhibits strong apical surface expression of Programmed Cell Death 1 Ligand 2 (PDCD1LG2, also known as PD-L2), another ligand for PDCD1 [57]. A single-cell RNA seq analysis generated from placental and decidual cells isolated from elective terminations of early pregnancy [58] provides compelling evidence that a variety of co-inhibitory ligands are expressed in those isolated cells defined as STB and that their corresponding receptors are present in either decidual or peripheral immune cells (especially T cells) or both (Figure 2A). These receptor/ligand combinations include VSIR/VSIR, LAG3/LGALS3, TIGIT/NECTIN2, CD200R1/CD200, and SIRPA/CD47, in addition to PDCD1/CD274 or PDCD1LG2 (Table 1).

Since the invasive, leading edge primitive STB of the implanting blastocyst cannot be studied in vivo in humans due to logistical and ethical limitations, we have generated an in vitro model of this primitive human STB using human embryonic stem cells (hESCs). In this model, hESCs or human induced pluripotent stem cells (hiPSCs) can be provoked by exposure to bone morphogenic protein 4 (BMP4) and inhibitors of transforming growth factor-beta (TGFB)/ACTIVIN and fibroblast growth factor-2 (FGF2) signaling to differentiate into cells that preferentially transcribe markers of trophoblast but not those characteristic of a diverse library of other tissues [60,61]. Trophoblast cells derived using these methods are referred to as BAP-differentiated or BAP cells. They acquire cell surface markers and secretory, transcriptional and functional profiles of STB and EVT and can be induced to progress preferentially down either developmental pathway [59]. These differentiated cells were noted, however, to have transcriptional profiles that, while consistent with trophoblast, differ from those of term cytotrophoblast cells syncytialized in vitro [59]. These and other data led to the hypothesis that these cells recapitulate early primitive trophoblast development [62]. RNA seq analysis has shown that most of the immune checkpoint receptor/ligand combinations seen in first trimester human placental specimens in vivo are also detectable in the in vitro-generated primitive STB (ESCd > 70; cell sheets larger than 70 μm in diameter) and term placenta-derived STB (PHTd in [61]) (Figure 2B) [59]. Some, such as VTCN1 (V-Set Domain Containing T Cell Activation Inhibitor 1) are highly expressed in primitive STB but barely detectable in term STB, suggesting gestational age dependence and a specific role in peri-implantation placental development, although the possibility exists that such up-regulation is a consequence of in vitro culture in absence of maternal tissue regulatory cues.

Emerging studies have indicated that immune checkpoints may be important in the maintenance of normal pregnancy. In human placental specimens obtained from women with preeclampsia or first trimester spontaneous abortions, CD200 expression is much lower than that in normal age-matched controls in which there is prominent expression at the apical surface of vSTB throughout gestation [63,64]. While checkpoint inhibition is a focus of therapy for cancer and autoimmune diseases [65,66], the role of therapeutic agents to modulate immune checkpoints in the control of immune interactions at the maternal–fetal interface remains incompletely studied.

### 2.4. A Role for STB-Derived Extracellular Vesicles

Extracellular vesicles (EV) are small membrane-coated particles [67,68] that are released by diverse cell types, including monocytes, endothelial cells, platelets, tumor cells and villous STB [67,69,70,71]. These include larger microparticles (MP; approximately 100–200 nm in diameter), which are essentially debris resulting from cellular apoptosis or activation, and smaller exosomes (typically 40–80 nm in diameter) actively generated and secreted from the exocytic compartment as an important part of cell–cell crosstalk [72,73].

Villous STB release of EVs is so robust that this trophoblast subtype is the primary source of circulating EVs in pregnant woman late in gestation. Increases in circulating microparticle levels and/or maternal response to these MPs have also been linked to preeclampsia [73]. The release of EVs, particularly MP, by villous STB is almost certainly an outcome of the syncytialization process itself as a result of the continuous incorporation of vCTB required for vSTB expansion and turnover [74]. Although apoptosis is characteristic of human vSTB, it is thought that the series of events that lead to apoptosis are initiated in vCTB and “passed” to Vstb [75]. EVs can signal through protein or lipid ligand-receptor interactions or via the micro-interfering RNAs (miRNAs) present in both soluble and EV-associated forms in various bodily fluids, including maternal serum [76] and amniotic fluid [77,78,79]. For instance, the checkpoint inhibitor molecules CD274 and CD276 (B7-H3) have been shown to be associated with exosomes derived from culture of first-trimester placental explants [80] and exosomes, microvesicles and apoptotic detritus has been shown to transfer microRNAs horizontally to local and distant cells (reviewed in [78]). This method of intercellular communication enables EVs to influence specific target cells, including, among others, peripheral and mucosal T cells, natural killer (NK) cells, B cells, monocytes/macrophages and dendritic cells [81,82]. EVs released into the maternal blood constitute a major signaling mechanism between fetus and mother throughout pregnancy [83]. Many cells at the maternal–fetal interface can release EVs and although it is difficult to determine the source of a particular EV in vivo, such intercellular communication has been shown to be a major immune modulator in the peri-implantation endometrium (reviewed in [84,85]).

### 2.5. Immune Modulation by Human Endogenous Retroviral Proteins

The human endogenous retroviruses (HERVs) are thought to have arisen from ancient infections of germ cells with introduction of viral DNA into the human genome in a manner that allowed for heritability. This has happened innumerable times throughout history so that approximately 8% of the human genome is retrovirus-derived [86]. Still, most of the coding sequences of the ERVs have undergone mutations that rendered their protein products non-functional. A few ERV-encoded genes, however, have retained expression of envelope (env)-derived proteins that have retained functional fusogenic properties [87,88,89,90]. The placentally expressed ERV-derived fusogens, syncytin 1 (*ERVW-1*), syncytin 2 (*ERVFRD-1*) and endogenous retrovirus 3-1 (*ERV3-1*) and the anti-fusogen *ERVRH48-1* (suppressyn/SUPYN) are some of the best studied of these proteins. These pro-fusogenic and anti-fusogenic placental ERV env proteins are likely quite tightly linked to trophoblast fusion, turnover and therefore microparticle release.

Many, if not all exogenous retroviruses, including human immunodeficiency virus, feline leukemia virus, human T cell leukemia virus, and others have immunosuppressive capabilities. It is perhaps not surprising, therefore, that many endogenized retroviruses have highly conserved immunosuppressive domains incorporated into the transmembrane portions their env proteins [91]. Human placental *ERVW-1*, *ERVFRD-1*, and *ERV3-1* each contain such domains and are potentially involved in immune modulation at the maternal–fetal interface. While the ERVs and their env immunosuppressive domains are believed to modulate the immune system in some manner, their mode of action on either the innate or adaptive systems remains unclear. Molecular mimicry, direct interaction with pattern recognition receptors, and superantigen activities have all been suggested as potential mechanisms [92,93]. In vitro assays have shown that the immunosuppressive and the pro-fusogenic activities of the placental ERV-derived proteins can be uncoupled. For example, isolated *ERVFRD-1* and *ERV3-1* env proteins are immunosuppressive, while ERVW-1 is not. Similarly, *ERVW-1* and *ERVFRD-1* are pro-fusogenic, but *ERV3-1* is not [94]. That said, the immunosuppressive effects of these molecules may be context specific, as immunosuppressive activities have been reported in vitro for both *ERVW-1* [95] and *sERVFRD-1* [96] when present on the surface of trophoblast derived extracellular vesicles. Despite derivation from distinct retroviral insertion events, two distinct *ERV* proteins (SYNA and SYNB) have assumed analogous fusogenic functions to *ERVW-1* and *ERVFRD-1* in the mouse [97].

There may be additional immunologic implications for the cooptation of these specific ERV env proteins in human placentation, with particular significance to the vSTB and its potential exposures to infectious particles circulating in maternal blood. *ERVH48-1* (suppressyn/SUPYN) is truncated so that it has lost its immunosuppressive domain and can be produced in both membrane-associated and soluble forms. *ERVRH48-1* blocks the profusogenic effects of *ERVW-1*, but not of *ERVFRD-1* in a dose-dependent fashion in in vitro models of cytotrophoblast fusion [98]. Importantly, the receptor for both ERVH48-1 and *ERVW-1* is called ASCT2/SLC1A5. Like most retroviral receptors, ASCT2/SLC1A5 has a physiologic function. It is widely expressed in a variety of cell types, where it acts as an amino acid transporter. It is of interest that it is also a receptor for large group of endogenous and exogenous, human and zoonotic viruses that share a phenomenon known as viral interference or superinfection resistance [99]. In this context, ASCT2/SLC1A5 is called the RD114/Type D retrovirus receptor. Families of often unrelated viruses can share a common receptor for cell entry. For the ASCT2/RD114/Type D receptor, these family members comprise the largest and most diverse of all viral interference groups and include baboon endogenous virus, type D primate retroviruses, avian reticuloendothelial viruses, the RD114 feline endogenous virus and, of course, the ERVW family, from which *ERVW-1* is derived [100]. Infection by one member of this interference family interferes with superinfection by other family members via alterations in the expression level of the shared host cell surface receptor and receptor glycosylation status; the latter affects its specificity in various cells [100]. SUPYN binding to ASCT2 alters receptor glycosylation and has been shown to inhibit interaction with the HERV-W env-derived *HERVW-1* [90,98]. A very similar effect has been reported for a protein product of the *fv4* gene in the *env* domain of murine leukemia virus (MuLV), which increases cellular resistance to superinfection with MuLV [100]. It is possible that in addition to its effects on *ERVW-1*, *ERVFRD-1*, and *ERV48-1* were involved in protection of placental superinfection by other HERVW or HERVF family members in the past and may still offer protection from placental infection by other known and unknown zoonotic interference family members.

### 2.6. Gestational Age-Specific Susceptibility of STB to Infection

In addition to potential immune modulatory functions, STB in the human placenta provides a relatively robust, but not insurmountable barrier to many pathogens, both bacterial (*Listeria monocytogenes* and *Toxoplasma gondii*) and viral (human cytomegalovirus (HCMV), herpes simplex virus-1 (HSV1), vesicular stomatitis virus (VSIV) and Zika virus (ZIKV)) [101,102,103,104,105,106]. Such resistance, however, may vary in efficacy by gestational age. To this latter point, several virus infections have been demonstrated to exert more dramatic fetal effects when infection occurs early in pregnancy [107,108,109,110]. While a comprehensive review of the mechanisms involved in trophoblast-specific responses to infection are beyond the specific purview of this review, we would like to highlight studies from our own work that suggest that the STB from early and late gestation may have inherent and diametrically opposed susceptibilities to a variety of pathogens. Using the stem cell-derived in vitro model of primitive trophoblast described earlier, we have determined the susceptibility of early human placenta to several viral pathogens.

Unlike term trophoblast cells that lack classic receptors for ZIKV entry and can mount a robust type III interferon (IFN) response [111], BAP-differentiated trophoblast cells express these receptors but have poor potential to respond to type III IFNs [112]. Each of these characteristics were consistent with our findings that the trophoblast derived from pluripotent cells that may be analogous to primitive trophoblast, and predominantly the STB patches within the cell colonies, were exquisitely sensitive to ZIKV infection and supported robust viral replication [112]. This was particularly true upon exposure to older Africa strains of the virus when compared to the newer Asian strains [110]. We found these results to be consistent with the hypothesis that the African strains of ZIKV may have exerted such devastating effects on the placenta that early pregnancy loss obfuscated the fetal effects noted after infection with the Asian strains of ZIKV first reported in Brazil in 2017. This primitive STB has a similar susceptibility to dengue virus infection and cytolysis (unpublished observations by Dr. Megan Sheridan), again contrasting with what is seen in term CTB cells syncytialized in vitro [111]. We have most recently begun to assess the potential for infection of primitive human trophoblast to severe acute respiratory syndrome coronavirus 2 (SARS Cov-2). Unlike term trophoblast in which known receptors for this virus, ACE2 and TMPRSS2, are expressed but on notably distinct placental cell subtypes [113], trophoblast cells differentiated in vitro from pluripotent stem cells appear to express both receptors on the STB (Dr. Jie Zhou, unpublished observations). The ability of these cells to support SARS Cov-2 infection and replication, plus ancillary effects of infection on cell survival are presently being determined, but receptor co-expression raises the possibility that, like ZIKV, the placenta of the peri-implantation human embryo may be susceptible to infection by SARS-Cov-2 and such infections could be causing undetected pre-clinical or very early clinical pregnancy losses.

One could hypothesize on an evolutionary advantage of such susceptibility to viral pathogens. Perhaps it is most adaptive for a woman who is infected in the peri-implantation period with ZIKV, dengue virus, SARS Cov-2, and potentially other viruses to suffer an early pregnancy loss in lieu of expending resources on a developing embryo while fighting maternal infection. Furthermore, early loss would avoid the possibility of more morbid (maternal and fetal) pregnancy loss at later stages of gestation or transmission to an infant.

## 3. Conclusions

The ability to develop syncytium from fusion of trophoblasts likely represents a critical evolutionary step in the development of the placenta in mammals and certain other viviparous invertebrates where placentation occurs [114,115]. This process has led to mechanisms whereby fetal protection from infection is balanced with modulation of potentially harmful maternal adaptive immune responses. The tightly regulated and complex process of generating and maintaining syncytium also represents a potential for early recognition of Danger signals and induction of innate immune responses. Enhanced understanding of this tissue and its functions may be the source of novel therapeutic agents in the prevention and treatment of abnormal pregnancy.

## Figures and Tables

**Table 1:** Placental inhibitory immune checkpoint receptor/ligand partners.

Receptors		Ligands	
Protein	Gene	Protein	Gene
PD1	*PDCD1*	PD-L1	*CD274*
		PD-L2	*PDCD1LG2*
CTLA4	*CTLA4*	B7-1	*CD80*
		B7-2	*CD86*
ICOS	*ICOS*	B7-H2	*ICOSLG*
		B7-H3	*CD276*
		B7-H4	*VTCN1*
B7-H5	*C10orf54*	B7-H5	*C10orf54* (*VSIR*)
TMIGD2	*TMIGD2*	B7-H7	*HHLA2*
TIM3	*HAVCR2*	GAL9	*LGALS9*
		CEACAM1	*CEACAM1*
		HMGB1	*HMGB1*
LAG3	*LAG3*	FGL1	*FGL1*
		GAL3	*LGALS3*
		CLEC4G	*CLEC4G*
TIGIT	*TIGIT*	CD155	*PVR*
		CD112	*PVRL2* (*NECTIN2*)
CD200R1	*CD200R1*	CD200	*CD200*
BTLA	*BTLA*	HVEM	*TNFRSF14*
SIRPα	*SIRPA*	CD47	*CD47*

## Data Availability

The data in presented Figure 2A in this study are openly available in http://data.teichlab.org (accessed on 1 January 2021) (maternal-fetal interface) provided at https://doi.org/10.1038/s41586-018-0698-6 (accessed on 1 January 2021) of reference number [58]. The data presented Figure 2B in this study are openly available in the Gene Expression Omnibus (GEO) database, www.ncbi.nlm.nih.gov/geo (accessed on 1 January 2021) (accession no. GSE73017) at https://doi.org/10.1073/pnas.1601630113 (accessed on 1 January 2021), reference number [59].
